# Four new species of the primitively segmented spider genus *Qiongthela* from Hainan Island, China (Mesothelae, Liphistiidae)

**DOI:** 10.3897/zookeys.911.48703

**Published:** 2020-02-12

**Authors:** Li Yu, Fengxiang Liu, Zengtao Zhang, Yan Wang, Daiqin Li, Xin Xu

**Affiliations:** 1 College of Life Sciences, Hunan Normal University, Changsha 410081, Hunan Province, China Hunan Normal University Changsha China; 2 State Key Laboratory of Biocatalysis and Enzyme Engineering, and Centre for Behavioural Ecology and Evolution (CBEE), School of Life Sciences, Hubei University, 368 Youyi Road, Wuhan 430062, Hubei Province, China Hubei University Wuhan China; 3 Yinggeling Nature Reserve, Baisha Li Autonomous County, 572800, China Yinggeling Nature Reserve Baisha Li Autonomous County China; 4 Department of Biological Sciences, National University of Singapore, 14 Science Drive 4, 117543, Singapore National University of Singapore Singapore Singapore

**Keywords:** Abdominal tergites, COI, genital morphology, taxonomy, trapdoor spiders

## Abstract

The primitively segmented spider genus *Qiongthela* Xu & Kuntner, 2015 consists of seven species that are distributed in Hainan Island, China and southern Vietnam. Of the seven species, five are known from Hainan Island. In this study, four more *Qiongthela* species collected from Hainan Island are diagnosed and described as new to science based on morphological characters: *Q. baoting***sp. nov.** (♂♀), *Q. qiongzhong***sp. nov.** (♂♀), *Q. sanya***sp. nov.** (♂♀), *Q. yinggezui***sp. nov.** (♂♀). To facilitate future identification, the GenBank accession codes of the DNA barcode gene, cytochrome c oxidase subunit I (COI), for all the type specimens are also provided.

## Introduction

As the sole extant lineage of the suborder Mesothelae, the primitively segmented spider family Liphistiidae is unique in having segmented plates on the abdomen (i.e., abdominal tergites) and in bearing spinnerets centrally on the ventral abdomen (Pocock 1892; Platnick and Gertsch 1976; Coddington and Levi 1991; Haupt 2003; Xu et al. 2015a, b). Its members live in underground burrows with a trapdoor, are long-lived, and have a limited dispersal ability (Bristowe 1976; Coddington and Levi 1991; Haupt 2003; Xu et al. 2015a, b). Liphistiidae is relatively species-poor, currently containing 131 described species in eight genera of two subfamilies, Liphistiinae Thorell, 1869 and Heptathelinae Kishida, 1923. It is constrained to East (China and Japan) and Southeast (Indonesia (Sumatra), Laos, Malaysia, Myanmar, Thailand, and Vietnam) Asia (Xu et al. 2015a, b, 2016; World Spider Catalog 2020). The subfamily Heptathelinae contains seven genera: *Ganthela* Xu & Kuntner, 2015 and *Sinothela* Haupt, 2003 limited to China only, *Heptathela* Kishida, 1923 and *Ryuthela* Haupt, 1982 restricted to Japan only, and the other three genera (*Qiongthela* Xu & Kuntner, 2015, *Songthela* Ono, 2000, and *Vinathela* Ono, 2000) occur in both China and Vietnam (Xu et al. 2015a, b, c, 2016, 2017a, b; World Spider Catalog 2020).

**Figure 1.**
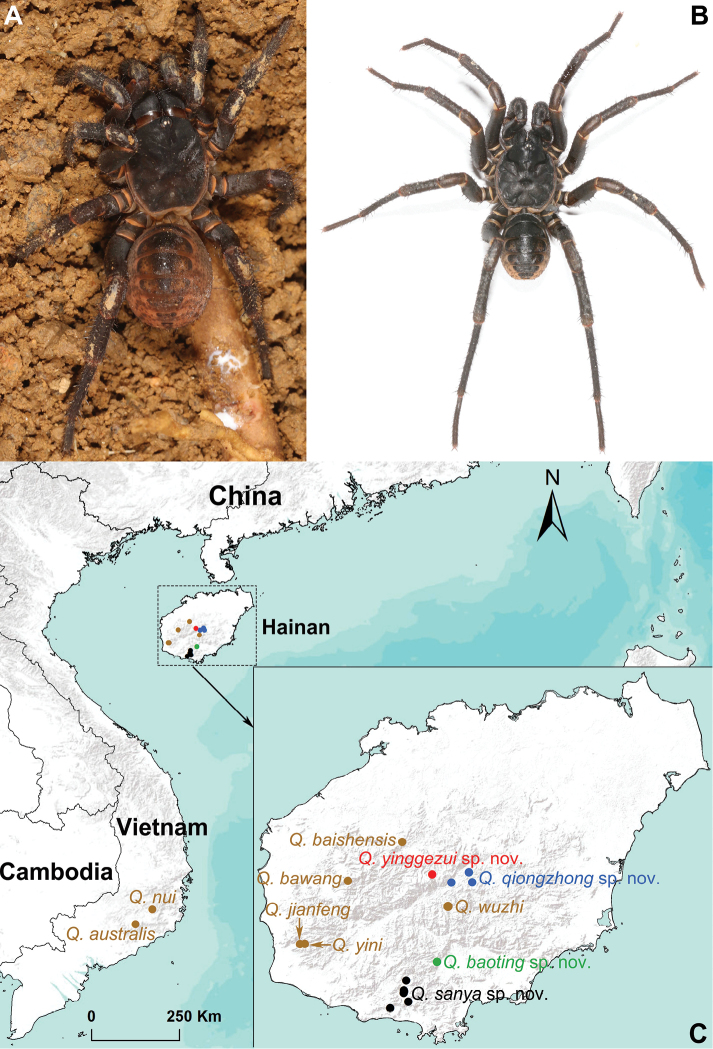
General somatic morphology of *Qiongthela baoting* sp. nov. and a map showing the type localities of seven known *Qiongthela* species and all sites of four new *Qiongthela* species in southern Vietnam and Hainan Island, China. **A** female (XUX–2017–196) **B** male (XUX–2017–195) **C** geographical map. Seven known species are indicated in brown solid circles, and four new species are indicated in red, blue, green, and black solid circles.

The genus *Qiongthela* was established by Xu and Kuntner in 2015 based on both morphological and molecular characters (Xu et al. 2015a, b). Until now, there are only seven named species, five of which are known from Hainan Island, China: *Q. baishensis* Xu, 2015, *Q. bawang* Xu, Liu, Kuntner & Li, 2017, *Q. jianfeng* Xu, Liu, Kuntner & Li, 2017, *Q. wuzhi* Xu, Liu, Kuntner & Li, 2017, and *Q. yini* Xu, Liu, Kuntner & Li, 2017 (Fig. 1C); the other two of which, *Q. australis* (Ono, 2002) and *Q. nui* (Schwendinger & Ono, 2011), are distributed in southern Vietnam (Fig. 1C) (Ono 2002; Schwendinger and Ono 2011; Xu et al. 2015a, b, 2017b; Word Spider Catalog 2020). In this study, we diagnosed and described four more new *Qiongthela* species collected from Hainan Island based on both male and female genital morphology. In addition, we also provided the COI sequences of the holotypes for facilitating future identification.

## Materials and methods

All specimens were collected from Hainan Island, China. All the type and voucher specimens are deposited at the College of Life Sciences, Hunan Normal University (HNU), Changsha, Hunan Province, China. We collected the spiders alive and fixed them in absolute ethanol if they were adults. For juvenile/subadult males, we took them back to the laboratory and reared them until they reached adulthood. We removed the right four legs of adults, preserved them in 100% ethanol and kept at –80°C for molecular work. We preserved the remains in 80% ethanol as vouchers for morphological identification and examination.

We examined and dissected the specimens using an Olympus SZ51 stereomicroscope. We cleaned the female genitalia in 10 mg/ml trypsase (Bomei Biotech Company, Hefei, Anhui, China) for at least 3 hours at the room temperature to dissolve soft tissues. We took the photos under the Olympus BX53 compound microscope using a digital camera CCD, and generated compound focussed images using Helicon Focus v6.7.1. All measurements were carried out under a digital camera MC170HD mounted on stereomicroscope Leica M205C and given in millimeters. Leg and palp measurements are given in the following order: leg total length (femur + patella + tibia + metatarsus + tarsus), palp total length (femur + patella + tibia + tarsus).

Abbreviations used are as follows: ALE = anterior lateral eyes; AME = anterior median eyes; BL = body length; CL = carapace length; Co = conductor; CT = contrategulum; CW = carapace width; E = embolus; OL = opisthosoma length; OW = opisthosoma width; PC = paracymbium; PLE = posterior lateral eyes; PME = posterior median eyes; RC = receptacular cluster; T = tegulum.

## Taxonomy

### Genus Qiongthela

Taxon classificationAnimaliaAraneaeLiphistiidae

Xu & Kuntner, 2015

510ADEA7-A3BA-5DED-BAFD-D4BB22FE36C3

#### Type species.

*Qiongthela baishensis* Xu, 2015

#### Diagnosis.

**Figure 2.**
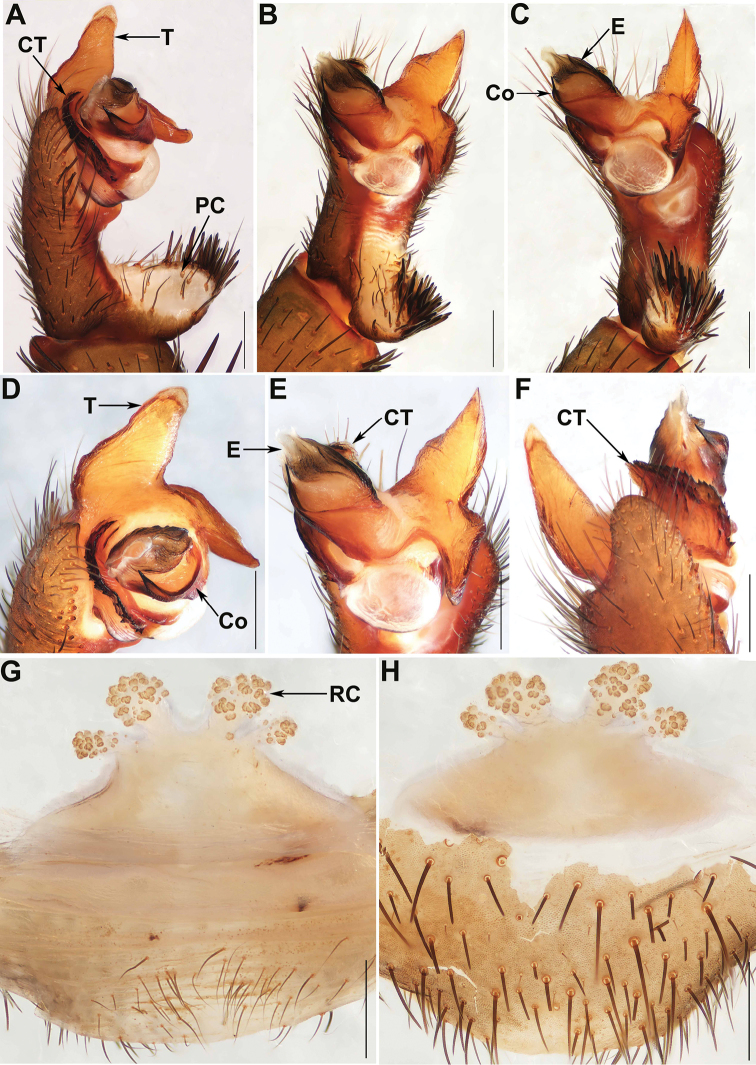
Male and female genital anatomy of *Qiongthela baoting* sp. nov. **A** palp prolateral view **B** palp ventral view **C** palp retrolateral view **D–F** palp distal view **G** vulva dorsal view **H** vulva ventral view. **A–F** XUX–2017–195 (holotype) **G–H** XUX–2017–196. Scale bars: 0.5 mm.

**Figure 3.**
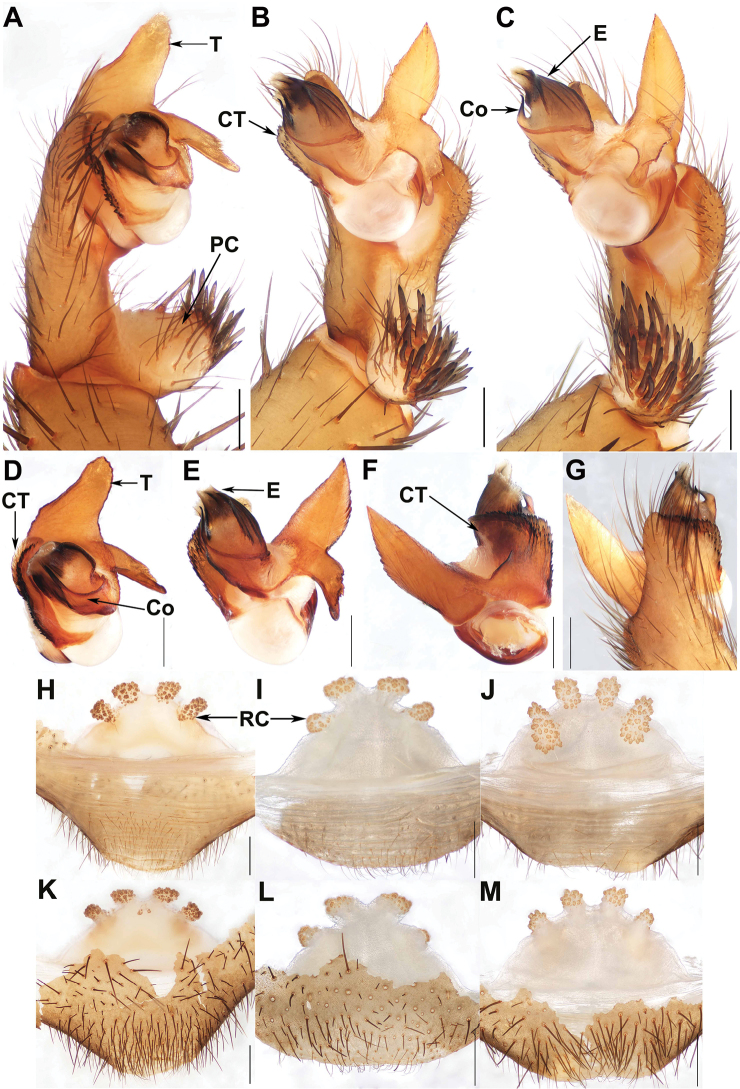
Male and female genital anatomy of *Qiongthela qiongzhong* sp. nov. **A** palp prolateral view **B** palp ventral view **C** palp retrolateral view **D–G** palp distal view **H–J** vulva dorsal view **K–M** vulva ventral view. **A–C, G** XUX–2017–156 (holotype) **D–F** XUX–2017–159 **H, K** XUX–2017–158 **I, L** XUX–2017–144 **J, M** XUX–2017–163. Scale bars: 0.5 mm.

**Figure 4.**
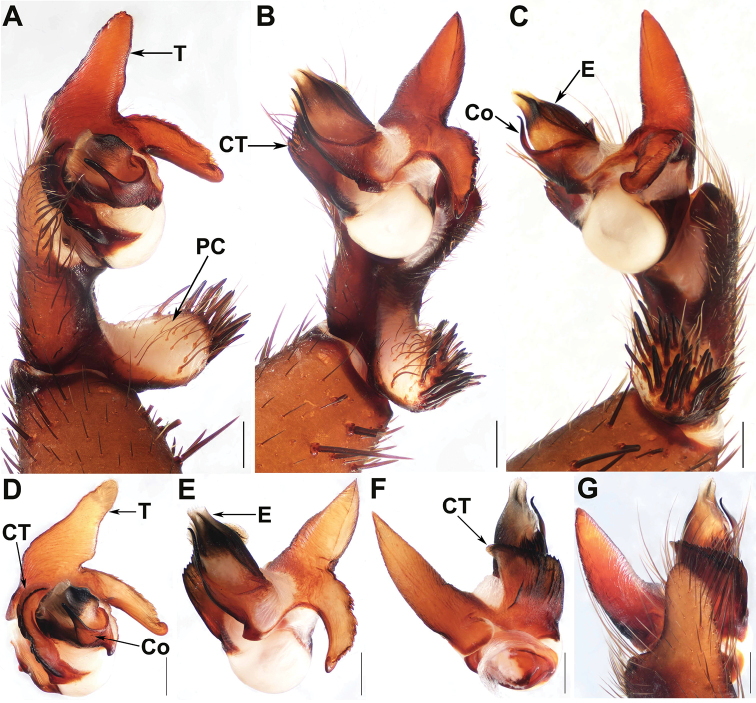
Male genital anatomy of *Qiongthela sanya* sp. nov. **A** palp prolateral view **B** palp ventral view **C** palp retrolateral view **D–G** palp distal view. **A–C, G** XUX–2017–219 (holotype) **D–F** XUX–2019–134. Scale bars: 0.5 mm.

**Figure 5.**
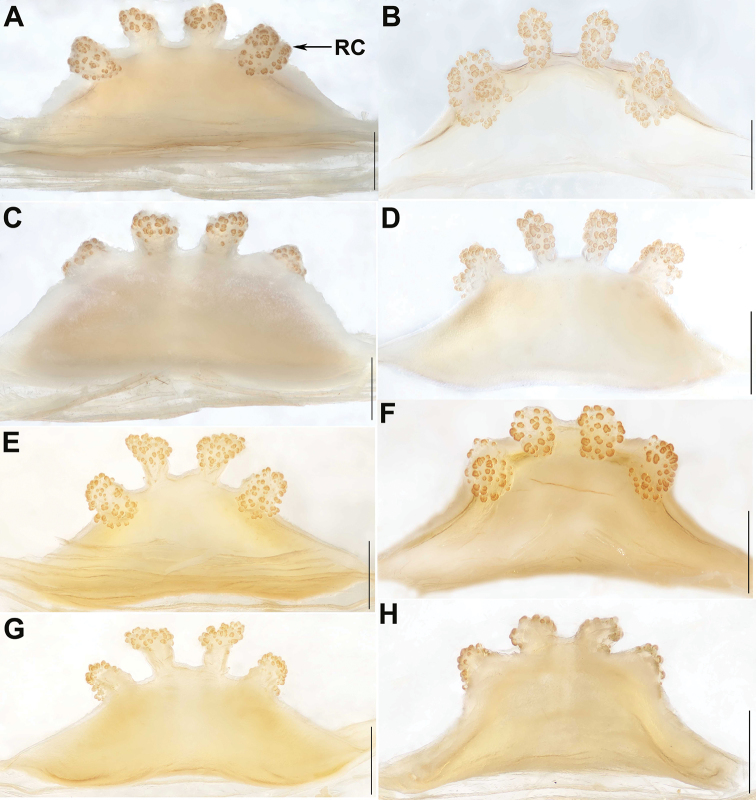
Female genital anatomy of *Qiongthela sanya* sp. nov. **A, B, E, F** vulva dorsal view **C, D, G, H** vulva ventral view. **A, C** XUX–2017–215 **B, D** XUX–2017–025 **E, G** XUX–2017–226 **F, H** XUX–2017–227. Scale bars: 0.5 mm.

**Figure 6.**
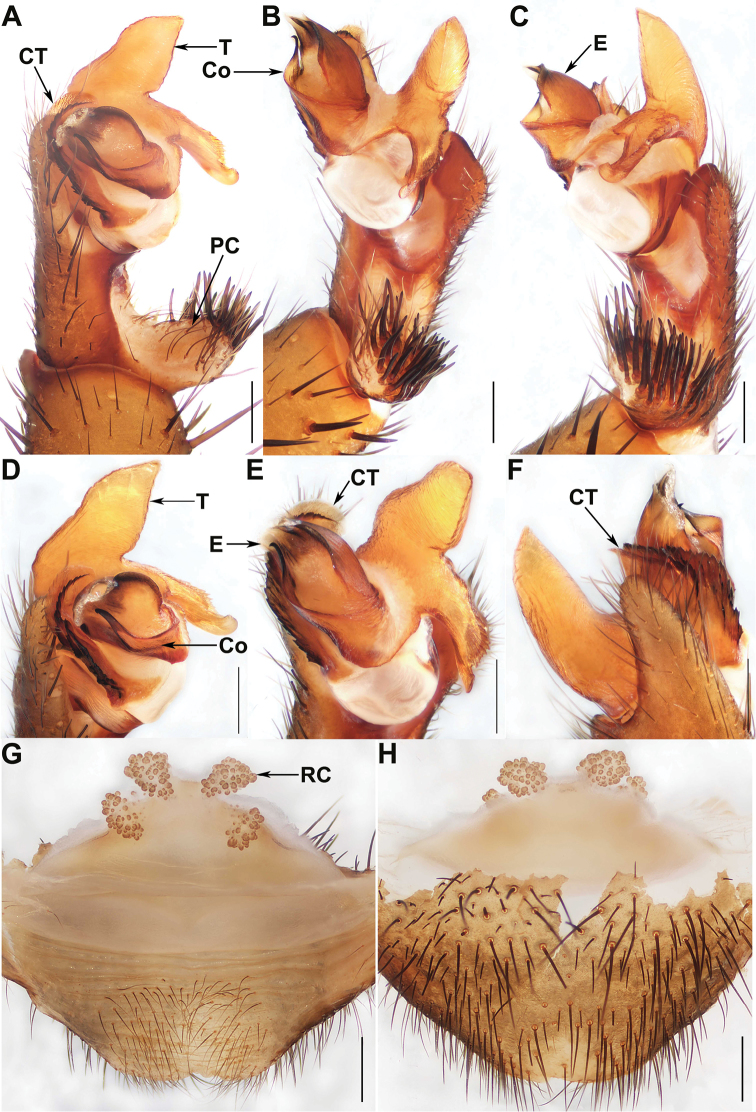
Male and female genital anatomy of *Qiongthela yinggezui* sp. nov. **A** palp prolateral view **B** palp ventral view **C** palp retrolateral view **D–F** palp distal view **G** vulva dorsal view **H** vulva ventral view. **A–F** XUX–2017–114 (holotype) **G–H** XUX–2017–121. Scale bars: 0.5 mm.

*Qiongthela* males can be distinguished from those of all other Heptathelinae genera by the blade-like conductor narrowing towards the tip (Figs 2A–D, 3A–E, 4A–G, 6A–E), and by the tegulum bearing two obvious apophyses (Figs 2A–E, 3A–E, 4A–E, 6A–E). *Qiongthela* females differ from those of all other Heptathelinae genera by two paired receptacular clusters with numerous granula (Fig. 5A–H) (Xu et al. 2017b).

#### Species composition.

*Q. australis* (Ono, 2002), *Q. baishensis* Xu, 2015, *Q. bawang* Xu, Liu, Kuntner & Li, 2017, *Q. jianfeng* Xu, Liu, Kuntner & Li, 2017, *Q. nui* (Schwendinger & Ono, 2011), *Q. wuzhi* Xu, Liu, Kuntner & Li, 2017, *Q. yini* Xu, Liu, Kuntner & Li, 2017.

#### Distribution.

China (Hainan), Vietnam.

### Qiongthela baoting sp. nov.

Taxon classificationAnimaliaAraneaeLiphistiidae

AD523BFA-F618-5B93-8D3A-C34CFE592D15

http://zoobank.org/C104261D-DBFB-4A70-84FD-5CF0BD15B82E

Figure 2

#### Type material.

**Holotype**: CHINA · 1 ♂; Hainan Province, Baoting County, Maogan Town, Zaye Village; 18.60°N, 109.57°E; alt. 410 m; 21 August 2017; D. Li, F.X. Liu and X. Xu leg.; XUX–2017–195 (matured on 25 August 2018 at HNU). **Paratype**: CHINA · 1 ♀; same data as for holotype; XUX–2017–196.

#### Diagnosis.

Male of *Q. baoting* sp. nov. can be distinguished from that of *Q. baishensis*, *Q. jianfeng*, *Q. nui*, *Q. wuzhi*, and the other three new species by the conductor with a pointed apex (Fig. 2A–D); from all the other *Qiongthela* species by the contrategulum with four edges distally (Fig. 2A, D), and by the marginal apophysis of the tegulum with a flake-like, semi-translucent apex (Fig. 2A, D, F). Female of *Q. baoting* sp. nov. differs from that of *Q. baishensis* and *Q. nui* by the base of the lateral receptacular clusters close to the inners, and by the genital stalks of the inners thicker than those of the laterals (Fig. 2G, H); from the other *Qiongthela* species by two paired receptacular clusters all along the anterior margin of the bursa copulatrix, with distinct genital stalks, and the inners larger than the laterals (Fig. 2G, H).

#### Description.

***Male*** (holotype, Fig. 1B). Carapace dark brown; opisthosoma light brown, with 12 dark brown tergites, close to each other, the first 2–7 larger than others, and the fourth largest; sternum narrow, much longer than wide; a few fine pointed hairs running over the ocular area; chelicerae with promargin of cheliceral groove bearing 9 denticles of variable size; legs with firm hairs and spines; 7 spinnerets. Measurements: BL 12.39, CL 6.17, CW 5.62, OL 6.44, OW 4.52; ALE > PLE > PME > AME; leg I 17.60 (5.15 + 1.65 + 3.82 + 4.28 + 2.69), leg II 16.95 (4.71 + 1.48 + 3.67 + 4.30 + 2.79), leg III 19.26 (4.56 + 1.31 + 3.73 + 6.04 + 3.63), leg IV 25.77 (6.52 + 1.61 + 5.32 + 7.68 + 4.64).

*Palp.* Cymbium with a short, thick projection dorsally (Fig. 2F); paracymbium unpigmented and unsclerotised prolaterally, with numerous setae at the tip (Fig. 2A, B). Contrategulum with an irregular dentate edge proximally and four edges distally: the inner edge sharp, very short; the middle two edges serrate, one towards the proximal portion of contrategulum, the other ended at the centre of the contrategulum; the outer edge short, smooth, slightly sclerotised (Fig. 2A, D, F). The marginal apophysis of tegulum long, wide basally, with a flake-like and semi-translucent apex distally (Fig. 2A, D), a proximally directed terminal apophysis of tegulum with smooth margin, narrowing to a slightly hooked apex (Fig. 2A–E). Conductor situated ventro-proximally on embolus, basal portion fused with embolus, distal free narrowing to a pointed apex (Fig. 2A–E). Embolus largely sclerotised, with a wide, flat opening of sperm duct distally (Fig. 2A, D, E).

***Female*** (Fig. 1A). Carapace dark brown; opisthosoma reddish brown, with 12 red-brown tergites, close to each other, the first 2–7 larger than the others, and the fourth largest; sternum narrow, nearly twice as long as wide; a few fine pointed hairs running over the ocular area; chelicerae robust with promargin of cheliceral groove containing 10 denticles of variable size; legs with firm hairs and spines; 7 spinnerets. Measurements: BL 16.35, CL 7.30, CW 6.12, OL 7.59, OW 6.25; ALE > PLE > PME > AME; palp 10.09 (3.66 + 1.01 + 2.60 + 2.82), leg I 11.78 (3.69 + 1.16 + 3.02 + 2.35 + 1.57), leg II 12.34 (3.94 + 1.44 + 2.69 + 2.58 + 1.70), leg III 10.99 (3.21 + 1.04 + 2.35 +2.90 + 1.47), leg IV 20.26 (5.85 + 1.93 + 4.17 + 5.45 + 2.86).

*Female genitalia.* Two pairs of receptacular clusters along the anterior margin of the bursa copulatrix, close to each other, the inner ones distinctly larger than the laterals, with genital stalks thicker than those of the laterals (Fig. 2G, H).

#### Etymology.

The species epithet, a noun in apposition, refers to the type locality.

#### Distribution.

Hainan (Baoting), China.

#### GenBank accession number.

Holotype (XUX–2017–195): MN911989.

### Qiongthela qiongzhong sp. nov.

Taxon classificationAnimaliaAraneaeLiphistiidae

C7B61FE6-1837-51AA-9689-929CF680C6B7

http://zoobank.org/09106528-8A15-461F-9042-3026C7C9E099

Figure 3

#### Type material.

**Holotype**: CHINA · 1 ♂; Hainan Province, Qiongzhong County, Yinggen Town, Chaocan Village; 19.08°N, 109.74°E; alt. 440 m; 15 August 2017; D. Li, F.X. Liu and X. Xu leg.; XUX–2017–156 (matured on 6 November 2017 at HNU). **Paratypes**: CHINA · 2 ♂♂, 2 ♀♀; same data as for holotype; XUX–2017–159, 161 (♂ matured on 6 November 2017 at HNU), XUX–2017–163 (♀ matured on 3 June 2018 at HNU), XUX–2017–158 · 6 ♂♂; Hainan Province, Qiongzhong County, Yinggen Town, Nabai Village; 19.03°N, 109.76°E; alt. 320 m; 14 August 2017; D. Li, F.X. Liu and X. Xu leg.; XUX–2017–148, 151, 154 (matured on 6 November 2017 at HNU), XUX–2017–149, 155 (matured on 10 November 2017 at HNU), XUX–2017–150 (matured on 14 January 2018 at HNU) · 1 ♀; Hainan Province, Qiongzhong County, Hongmao Town, Caohui Village; 19.03°N, 109.65°E; alt. 345 m; 14 August 2017; D. Li, F.X. Liu and X. Xu leg.; XUX–2017–144 · 2 ♂♂, 2 ♀♀; same locality as for holotype; 19.08°N, 109.74°E; alt. 420 m; 17 August 2019; D. Li, F.X. Liu, X. Xu and L. Yu leg.; XUX–2019–111 (♂, matured on 16 October 2019 at HNU), XUX–2019–112 (♂, matured on 23 October 2019 at HNU), XUX–2019–108, 109.

#### Diagnosis.

Males of *Q. qiongzhong* sp. nov. resemble those of *Q. yinggezui* sp. nov., but can be distinguished from those of the latter by the marginal apophysis of the tegulum with a blunt apex (Fig. 3A, D); from *Q. baoting* sp. nov. by the tegulum marginal apophysis with a non-translucent apex (Fig. 3A, D), by the contrategulum with two edges distally (Fig. 3A, D), and by the cymbial projection long and thin (Fig. 3G); from *Q. australis* by the conductor with a slightly bent apex (Fig. 3C, E, G), and by the contrategulum lacking beak-like extension (Fig. 3F); from *Q. jianfeng* by the terminal apophysis of the tegulum abruptly narrowed distally (Fig. 3A–C); from *Q. nui*, *Q. sanya* sp. nov., and *Q. wuzhi* by the marginal apophysis of the tegulum with a blunt apex (Fig. 3A, D). Females of *Q. qiongzhong* sp. nov. can be distinguished from those of *Q. bawang* and *Q. jianfeng* by the receptacular clusters with indistinct genital stalks (Fig. 3H, J); from those of *Q. baishensis*, *Q. baoting* sp. nov., *Q. nui*, *Q. yini*, and *Q. wuzhi* by the similar-sized receptacular clusters or the laterals slightly larger than the inners (Fig. 3H–M).

#### Description.

***Male*** (holotype). In alcohol carapace light reddish brown; opisthosoma light brown, with brown 12 tergites, close to each other, the first 2–7 larger than others, and the fourth largest; sternum narrow, nearly twice as long as wide; a few fine pointed hairs running over the ocular area; chelicerae with promargin of cheliceral groove containing 10 denticles of variable size; legs with firm hairs and spines; 7 spinnerets. Measurements: BL 13.34, CL 6.13, CW 5.61, OL 7.17, OW 5.50; ALE > PLE > PME > AME; leg I 16.64 (4.88 + 1.54 + 4.04 + 4.03 + 2.15), leg II 16.25 (4.62 + 1.32 + 3.60 + 4.51 + 2.20), leg III 17.39 (4.57 + 1.34 + 3.48 + 5.32 + 2.68), leg IV 22.50 (6.06 + 1.49 + 5.11 + 6.81 + 3.04).

*Palp.* Cymbium with a long, thin projection dorsally (Fig. 3G); paracymbium unpigmented and unsclerotised prolaterally, with numerous setae at the tip (Fig. 3A, B). Contrategulum with a proximally irregular dentate edge and two distal edges: the inner one irregularly dentate, and the outer one sharp, semi-translucent (Fig. 3A, D–F). The marginal apophysis of tegulum with a blunt, slightly dentate apex distally, a proximally directed terminal apophysis of tegulum with several denticles and an abruptly narrowed and slightly hooked apex (Fig. 3A–E). Conductor situated ventro-proximally on embolus, the basal portion fused with embolus, distal free, narrowing to a slightly bent apex (Fig. 3A–C, E). Embolus largely sclerotised, retrolaterally with numerous longitudinal ribs, and with a wide, flat sperm duct opening distally (Fig. 3A, D, F).

***Female*** (XUX–2017–158). In alcohol carapace reddish brown; opisthosoma brown; opisthosoma with 12 dark brown tergites, separated from each other, the first 2–7 larger than others, and the fourth largest; sternum narrow, nearly twice as long as wide; a few fine pointed hairs running over the ocular area; chelicerae with promargin of cheliceral groove containing 10 strong denticles of variable size; legs with firm hairs and spines; 7 spinnerets. Measurements: BL 16.59, CL 6.93, CW 6.18, OL 9.48, OW 7.83; ALE > PLE > PME > AME; palp 11.02 (3.80 + 1.20 + 2.86 + 3.16), leg I 13.69 (4.53 + 1.52 + 2.99 + 2.87 + 1.77), leg II 12.61 (3.80 + 1.39 + 2.77 + 2.78 + 1.87), leg III 12.06 (3.71 + 1.04 + 2.46 + 3.27 + 1.58), leg IV 20.31 (6.08 + 1.67 + 4.24 + 5.55 + 2.77).

*Female genitalia.* Two pairs of receptacular clusters along the anterior margin of the bursa copulatrix, receptacular clusters similar size or the inner ones slightly smaller than the lateral ones, with indistinct genital stalks (Fig. 3H–M).

#### Variation.

Males and females vary in body size. The range of measurements in males (*N* = 11): BL 12.43–17.24, CL 5.99–7.80, CW 5.61–7.12, OL 6.52–9.52, OW 4.67–7.02; females (*N* = 5): BL 9.93–16.59, CL 4.91–7.38, CW 4.25–6.51, OL 4.93–9.48, OW 3.48–7.83. In addition, female genitalia show considerable intraspecific variation: the receptacular clusters vary in shape: triangular (Fig. 3H, J), or oval (Fig. 3I, L); the ventral side of the bursa copulatrix with two small granula (Fig. 3K); the posterior part of genital area arched (Fig. 3I, L), or with a slightly notch in the middle (Fig. 3J, M).

#### Etymology.

The species epithet, a noun in apposition, refers to the type locality.

#### Distribution.

Hainan (Qiongzhong), China.

#### GenBank accession number.

Holotype (XUX–2017–156): MN911987.

### Qiongthela sanya sp. nov.

Taxon classificationAnimaliaAraneaeLiphistiidae

59443834-E7F3-5A14-B719-A625AC9C61F3

http://zoobank.org/F46F043A-D2BD-4BE0-B24D-771C53F26BDB

Figures 4 , 5

#### Type material.

**Holotype**: CHINA · 1 ♂; Hainan Province, Sanya City, Tianya District, Zhaka Village; 18.50°N, 109.41°E; alt. 240 m; 22 August 2017; D. Li, F.X. Liu and X. Xu leg.; XUX–2017–219. **Paratypes**: CHINA · 1 ♀; same data as for holotype; XUX–2017–218 · 1 ♀; Hainan Province, Sanya City, Heshangling; 18.35°N, 109.32°E; alt. 130 m; 1 August 2017; D. Li, F.X. Liu, Z.T. Zhang and X. Xu leg.; XUX–2017–025 · 1 ♂, 2 ♀♀; Hainan Province, Sanya City, Tianya District, Baoqian Village; 18.39°N, 109.42°E; alt. 195 m; 22 August 2017; D. Li, F.X. Liu and X. Xu leg.; XUX–2017–205 (♂ matured on 29 October 2017 at HNU), XUX–2017–202, 209 · 1 ♂, 10 ♀♀; Hainan Province, Sanya City, Tianya District, Nandao Farm, Sanmudong; 18.44°N, 109.40°E; alt. 200 m; 21 August 2019; D. Li, F.X. Liu, X. Xu and L. Yu leg.; XUX–2019–134 (♂ matured on 2 October 2019 at HNU), XUX–2019–136 to 137H · 9 ♀♀; Hainan Province, Sanya City, Tianya District, Nandao Farm, Haiyan Group; 18.45°N, 109.40°E; alt. 215 m; 22 August 2017; D. Li, F.X. Liu and X. Xu leg.; XUX–2017–214 to 217, XUX–2017–221, 222, XUX–2017–225 to 227 · 1 ♀; Hainan Province, Sanya City, Tianya District, between Hongxing Farm and Zhaka Village; 18.50°N, 109.41°E; alt. 235 m; 22 August 2017; D. Li, F.X. Liu and X. Xu leg.; XUX–2017–220 · 1 ♀; Hainan Province, Sanya City, Tianya District, Nandao Farm, Haiying Group; 18.43°N, 109.39°E; alt. 200 m; 21 August 2019; D. Li, F.X. Liu, X. Xu and L. Yu leg.; XUX–2019–131.

#### Diagnosis.

Males of *Q. sanya* sp. nov. can be distinguished from those of *Q. baoting* sp. nov. by the longer tegulum marginal apophysis with a non-translucent apex (Fig. 4A, D), and by the conductor with a bent apex (Fig. 4C, E, F, G); from those of the other *Qiongthela* species by the conductor base with a triangular apophysis ventrally (Fig. 4A–E). Females of *Q. sanya* sp. nov. can be distinguished from *Q. australis*, *Q. yini* and *Q. yinggezui* sp. nov. by the inner receptacular clusters smaller than the lateral ones (Fig. 5A–H); from those of the other *Qiongthela* species by the inner receptacular clusters along the anterior margin of the bursa copulatrix, the laterals located slightly on the dorsal wall of the bursa copulatrix, and by the trapezoidal bursa copulatrix (Fig. 5A–H).

#### Description.

***Male*** (holotype). In alcohol carapace reddish dark; opisthosoma brown, with 12 reddish dark tergites, close to each other, the first 2–7 larger than others, and the fourth largest; sternum narrow, nearly twice as long as wide; a few fine pointed hairs running over the ocular area; chelicerae with promargin of cheliceral groove containing 9 denticles of variable size; legs with firm hairs and spines; 7 spinnerets. Measurements: BL 13.40, CL 6.47, CW 5.87, OL 6.80, OW 5.20; ALE > PLE > PME > AME; leg I 22.06 (6.30 + 1.62 + 5.26 + 5.97 + 2.90), leg II 20.17 (5.16 + 1.50 + 4.81 + 5.77 + 2.94), leg III 22.02 (5.65 + 1.62 + 4.41 + 6.83 + 3.52), leg IV 28.13 (7.15 + 1.87 + 6.00 + 8.93 + 4.17).

*Palp.* Cymbium with a short projection dorsally (Fig. 4G); prolateral side of paracymbium unpigmented and unsclerotised, with numerous setae at the tip (Fig. 4A–C). Contrategulum with two distal edges: the inner one strongly dentate, and the outer one smooth, sharp, semi-translucent (Fig. 4A, D, F). Tegulum with a long, pointed, distally directed marginal apophysis, the proximally directed terminal apophysis with a dentate margin and continuously narrowing to a rounded, hooked apex (Fig. 4A–E). Conductor situated ventro-proximally on embolus, fused with embolus at the basal portion, distal free narrowing to a bent apex (Fig. 4B, C, E–G); conductor base with a triangular apophysis ventrally (Fig. 4A–E). Embolus largely sclerotised, with a wide, flat sperm duct opening distally, retrolaterally with numerous longitudinal ribs (Fig. 4B, C, E).

***Female*** (XUX–2017–215). In alcohol carapace reddish dark; opisthosoma dark brown, with 12 reddish dark tergites, close to each other, the first 2–7 larger than others, and the fourth largest; sternum narrow, much longer than wide; a few fine pointed hairs running over the ocular area; chelicerae with promargin of cheliceral groove containing 10 strong denticles of variable size; legs with firm hairs and spines; 7 spinnerets. Measurements: BL 25.50, CL 11.95, CW 10.97, OL 13.00, OW 10.75; ALE > PLE > PME > AME; palp 18.86 (6.35 + 2.20 + 4.73 + 5.58), leg I 23.55 (8.14 + 3.11 + 5.51 + 4.44 + 2.35), leg II 21.33 (7.25 + 2.68 + 4.87 + 4.25 + 2.28), leg III 22.43 (7.19 + 2.50 + 4.99 + 4.98 + 2.78), leg IV 34.17 (10.27 + 3.17 + 7.11 + 9.07 + 4.55).

*Female genitalia.* The inner receptacular clusters along the anterior margin of the bursa copulatrix, the lateral ones located slightly on the dorsal wall of the bursa copulatrix; the inner ones smaller than the lateral ones, with short or long genital stalks. The bursa copulatrix trapezoidal (Fig. 5A–H).

#### Variation.

Males and females vary in body size. The range of measurements in males (*N* = 3): BL 13.40–15.01, CL 6.47–7.21, CW 5.87–6.53, OL 6.16–7.53, OW 4.47–5.20; females (*N* = 25): BL 15.41–27.74, CL 7.32–14.14, CW 6.23–11.59, OL 7.33–13.49, OW 5.70–11.84. In addition, female genitalia show intraspecific variation: the inner pair of the receptacular clusters along the anterior margin of the bursa copulatrix upward, with short or long genital stalks (Fig. 5A–E, G), or clusters toward the dorsal margin (Fig. 5F, H).

#### Etymology.

The species epithet, a noun in apposition, refers to the type locality.

#### Distribution.

Hainan (Sanya), China.

#### GenBank accession number.

Holotype (XUX–2017–219): MN911990.

### Qiongthela yinggezui sp. nov.

Taxon classificationAnimaliaAraneaeLiphistiidae

9F1E4675-5661-50E2-924B-4D189E0AC0ED

http://zoobank.org/72CEC4E7-BE97-4E42-8F90-559DAA2AC067

Figure 6

#### Type material.

**Holotype**: CHINA· 1 ♂; Hainan Province, Qiongzhong County, 3.7 Km to Yinggezui; 19.07°N, 109.55°E; alt. 710 m; 11 August 2017; D. Li, F.X. Liu, Z.T. Zhang and X. Xu leg.; XUX–2017–114 (matured on 29 September 2017 at HNU)**. Paratypes**: CHINA · 3 ♀♀; same data as for holotype; XUX–2017–115, 116, 121.

#### Diagnosis.

Male of *Q. yinggezui* sp. nov. differs from that of *Q. australis* by the conductor base wide and with a bent apex (Fig. 6A–F), and by the shorter paracymbium (Fig. 6A); from *Q. nui* by the embolus with a smooth surface retrolaterally (Fig. 6B, C, E); from *Q. baoting* sp. nov. by the cymbium with an elongated projection (Fig. 6F), and by the conductor with a bent apex (Fig. 6B–E); from *Q. jianfeng*, *Q. qiongzhong* sp. nov. and *Q. sanya* sp. nov. by the scutiform marginal apophysis of the tegulum thick basally and pointed distally (Fig. 6A–F), and by the embolus with a smooth surface retrolaterally (Fig. 6B, C, E). Females of *Q. yinggezui* sp. nov. can be distinguished from those of *Q. australis* by the similar-sized receptacular clusters, and the lateral ones slightly located on the dorsal wall of the bursa copulatrix (Fig. 6G); from *Q. yini* by the receptacular clusters with more granula (Fig. 6G, H); from *Q. sanya* sp. nov. by the lack of genital stalks (Fig. 6G, H); from those of the other *Qiongthela* species by the inner receptacular clusters situated at the anterior margin of bursa copulatrix, the lateral pair located on the dorsal wall of the bursa copulatrix (Fig. 6G, H).

#### Description.

**Male** (holotype). In alcohol carapace light reddish brown; opisthosoma light brown, with 12 brown tergites, separated from each other, the first 2–7 larger than others, and the fourth largest; sternum narrow, nearly twice as long as wide; a few fine pointed hairs running over the ocular area; chelicerae with promargin of cheliceral groove containing 9 denticles of variable size; legs with firm hairs and spines; 7 spinnerets. Measurements: BL 13.60, CL 5.99, CW 6.30, OL 7.29, OW 5.48; ALE > PLE > PME > AME; leg I 17.44 (4.82 + 1.55 + 4.10 + 4.51 + 2.46), leg II 17.50 (4.73 + 1.43 + 3.97 + 4.80 + 2.57), leg III 16.73 (4.68 + 1.36 + 2.40 + 5.59 + 2.70), leg IV 25.19 (6.52 + 1.62 + 5.28 + 8.00 + 3.77).

*Palp.* Cymbium with an elongated projection dorsally (Fig. 6F); prolateral side of paracymbium unpigmented and unsclerotised, with numerous setae at the tip (Fig. 6A, B). Contrategulum with a proximally irregular dentate edge and two distal edges: the inner one dentate, the outer one smooth, sharp, semi-translucent, fused with the inner one at the middle portion of contrategulum (Fig. 6A, D–F). The marginal apophysis of tegulum long, pointed with a sharp apex, a proximally directed terminal apophysis with finely dentate margin and continuously narrowing to a rounded, hooked apex (Fig. 6A–E). Conductor situated ventro-proximally on embolus, fused with embolus at the basal portion, distal free narrowing to a bent apex (Fig. 6A–C, E). Embolus largely sclerotised, with a wide, flat sperm duct opening, and with a smooth surface retrolaterally (Fig. 6A–E).

**Female** (XUX–2017–121). In alcohol carapace reddish brown; opisthosoma brown; opisthosoma with 12 tergites, closed to each other, the first 2–7 larger than others, and the fourth largest; sternum narrow, more than twice the width; a few fine pointed hairs running over the ocular area; chelicerae with promargin of cheliceral groove containing 10 denticles of variable size; legs with firm hairs and spines; 7 spinnerets. Measurements: BL 14.76, CL 7.03, CW 6.39, OL 7.82, OW 6.03; ALE > PLE > PME > AME; palp 13.30 (5.30 + 1.23 + 2.85 + 3.91), leg I 14.35 (4.84 + 1.54 + 3.21 + 2.95 + 1.82), leg II 12.72 (3.54 + 1.24 + 2.97 + 2.99 + 1.98), leg III 13.78 (4.20 + 1.28 + 2.60 + 3.71 + 1.99), leg IV 20.21 (5.29 + 1.38 + 4.52 + 5.78 + 3.24).

*Female genitalia.* Two paired of the similar-sized receptacular clusters, the inner ones along the anterior margin of the bursa copulatrix, and the lateral ones located slightly on the dorsal wall of the bursa copulatrix, without genital stalks (Fig. 6G, H).

#### Variation.

Females vary in body size. The range of measurements in females (*N* = 3): BL 11.51–14.76, CL 4.68–7.03, CW 4.54–6.39, OL 5.54–7.82, OW 4.32–6.03.

#### Etymology.

The species epithet, a noun in apposition, refers to the type locality.

#### Distribution.

Hainan (Yinggezui), China.

#### GenBank accession number.

Holotype (XUX–2017–114): MN911988.

## Supplementary Material

XML Treatment for Qiongthela

XML Treatment for Qiongthela baoting

XML Treatment for Qiongthela qiongzhong

XML Treatment for Qiongthela sanya

XML Treatment for Qiongthela yinggezui
